# Clinical Significance of Serum Haptoglobin and Protein Disulfide-Isomerase A3 in the Screening, Diagnosis, and Staging of Colorectal Cancer

**DOI:** 10.3389/fphar.2022.935500

**Published:** 2022-07-04

**Authors:** Yajin Niu, Jun Xue, Xueliang Wu, Ming Qu, Likun Wang, Weizheng Liang, Tian Li

**Affiliations:** ^1^ Graduate School, Hebei North University, Zhangjiakou, China; ^2^ Department of General Surgery, The First Affiliated Hospital of Hebei North University, Zhangjiakou, China; ^3^ Central Laboratory, The First Affiliated Hospital of Hebei North University, Zhangjiakou, China; ^4^ School of Basic Medicine, Fourth Military Medical University, Xi’an, China

**Keywords:** colorectal cancer, haptoglobin, protein disulfide-isomerase A3, screening, diagnosis, staging, sequencing

## Abstract

**Objective:** This study aims to explore the clinical significance of haptoglobin (HP) and protein disulfide-isomerase A3 (PDIA3) in human serum in the screening, diagnosis and staging of colorectal cancer (CRC), and to provide novel screening approaches featuring high specificity, sensitivity, and accuracy for early screening and diagnosis of clinical colorectal cancer.

**Methods:** 88, 77, and 36 blood specimens were respectively harvested from colorectal cancer patients, colorectal polyp patients, and normal subjects (the health examination) who requested medical assistance from our hospital between Oct2019 and February 2022. The serum contents of HP and PDIA3 in each sample were determined through an enzyme linked immunosorbent assay (ELISA). This step was taken to analyze the differences among different specimen groups in terms of the serum contents of HP and PDIA3, to analyze the relationships between the expression levels of HP and PDIA3 and the pathological characteristics of colorectal cancer, and to explore the critical role of HP and PDIA3 in the screening, diagnosis, and staging of colorectal cancer.

**Results:** Serum contents of HP and PDIA3 were higher in colorectal cancer patients, with statistical differences (*p* < 0.05), than those in the colonic polyp patients and healthy subjects. Receiver operating characteristic (ROC) curve demonstrated that the cut-offs of HP and PDIA3 serum contents indicating colorectal cancer were 149 ug/ml and 66 ng/ml respectively. The individually and jointly tested AUCs of HP (0.802) and PDIA3 (0.727) were higher than those of serum CEA and CA199, the sensitivity and specificity of HP were 64.8 and 91.2%, the sensitivity and specificity of PDIA3 were 65.9 and 71.7%. Moreover, the contents of HP and PDIA3 increased alongside disease progression, with differences (*p* < 0.05).

**Conclusion:** Our research indicated that joint testing of HP and PDIA3 was of reference value for progressive stage and reliable biological indicators of colorectal cancer screening.

## Introduction

Colorectal cancer (CRC) is one of the most common malignancies worldwide (Karpiński et al., 2022; Andrei et al., 2021; Ocvirk and O'Keefe, 2021). As per the *2020 World Cancer Report* issued by the International Agency for Research on *Cancer* (IARC) in 2020, the number of new cases and deaths of colorectal cancer worldwide would exceed 1.93 million and 0.93 million respectively by 2020 (Mattiuzzi and Lippi, 2020). A statistical report on United States cases of colorectal cancer in 2020 (Siegel et al., 2020) indicated that the US predicted 147,950 new cases and 53,200 deaths in 2020xx. Since the 1970s, the incidence and mortality of colorectal cancer in the US have both experienced a sustained decline as the target group of colorectal cancer screening has gradually shifted from high-risk population to healthy subjects. In the US, the ratio of colonoscopy screening increased from 20% in 2000 to 61% in 2018. In sharp contrast to the US reality, the incidence and mortality of colorectal cancer have both surged in China. It was estimated that China would have 550,000 new cases and 280,000 deaths of colorectal cancer in 2020 (Wang, 2021). Based on the comparison of relevant data between China and the US, early screening is essential to the diagnosis and treatment of colorectal cancer. However, both early diagnosis and screening of colorectal cancer remain subpar in China.

In 90% of colorectal cancer cases, disease progression follows an established pattern, from adenoma to tumor and it usually takes five to 10 years for a benign lesion to progress into a malignant tumor, which provides a substantial window for the early screening of colorectal cancer (Dekker et al., 2019). Meanwhile, the prognosis of colorectal cancer is intricately linked to early diagnosis and treatment. The 5-years survival rate of colorectal cancer would decrease from 91% in the early stage to around 10% in the advanced stage (Siegel et al., 2020). Numerous studies suggest that the nature of colorectal cancer, as a special biological behavior, determines that the prognosis of such patients can be improved through early screening, diagnosis, and treatment. Currently, the commonly used screening methods in China include digital rectal examination, fecal occult blood test, serum tumor marker test, and other testing methods. Moreover, invasive testing includes electronic colonoscopy and other screening approaches (Shaukat et al., 2021). However, these screening methods cannot satisfactorily achieve the need for screening due to their lack of sensitivity and specificity as well as poor patient compliance and financial circumstances. Therefore, the establishment of screening methods having high sensitivity and specificity, good compliance of the screening target group, and cost acceptable to the target population is essential.

**FIGURE 1:**
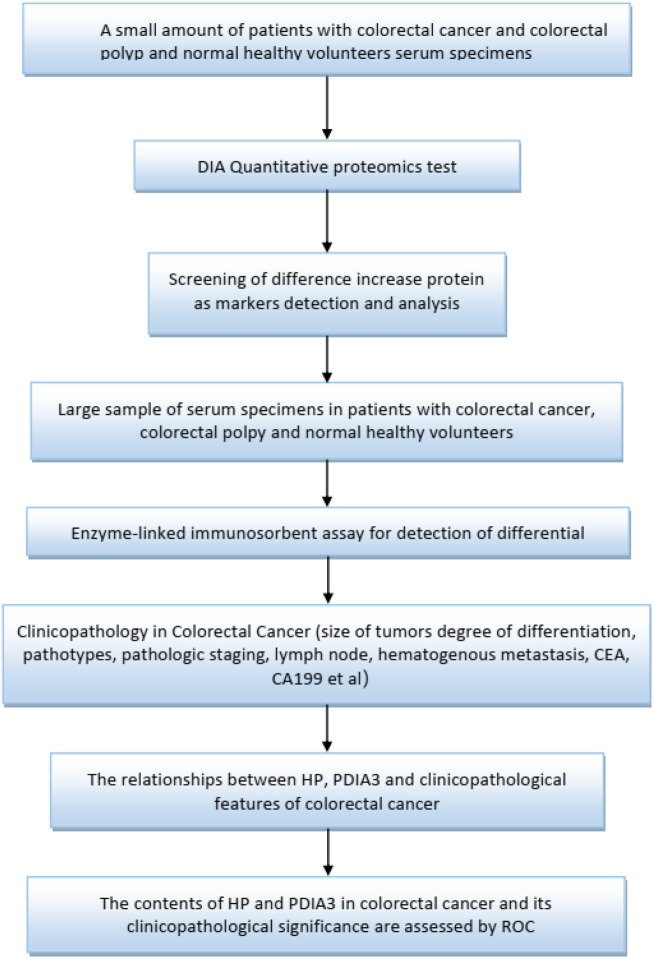
Flow chart of study design.

In this study, small samples were utilized to conduct date-independent acquisition (DIA), quantitative proteomics testing to select haptoglobin (HP) and protein disulfide-isomerase A3 (PDIA3) with significant differences for function enrichment. The test was followed by verification through larger samples. HP is an acute-phase protein with a role in the neutralization and clearance of free heme. Iron has tremendous potential for initiating vascular oxidation, inflammation and exacerbating coronary atherosclerosis. Hp genotype has been linked as a prognostic biomarker of acute myocardial infarction (Chapelle et al., 1982; Suleiman et al., 2005), heart failure (Holme et al., 2009; Haas et al., 2011), restenosis (Moussa et al., 2014) and cardiac transplant rejection (Shen et al., 2012; Shen et al., 2015). The increased understanding of Hp as a biomarker has provided new insights into the mechanisms of inflammation after cardiac injury and support the concept that Hp is not only an important antioxidant in vascular inflammation and atherosclerosis, but also an enhancer of inflammation in cardiac transplant (Graves and Vigerust, 2016). It is known that oxidative stress is a risk factor for cancer development. A common functional haptoglobin (Hp) polymorphism, originating from a duplication of a gene segment spanning over two exons, results in three distinct phenotypes with different anti-oxidative capacities: Hp1-1, Hp1-2, and Hp2-2 (Kaiser et al., 2020). The catalysis of disulphide (SS) bonds is the most important characteristic of protein disulphide isomerase (PDI) family. Catalysis occurs in the endoplasmic reticulum, which contains many proteins, most of which are secretory in nature and that have at least one s-s bond. Protein disulphide isomerase A3 (PDIA3) is a member of the PDI family that acts as a chaperone. PDIA3 is highly expressed in response to cellular stress, and also intercept the apoptotic cellular death related to endoplasmic reticulum (ER) stress, and protein misfolding. PDIA3 expression is elevated in almost 70% of cancers and its expression has been linked with overall low cell invasiveness, survival and metastasis (Mahmood et al., 2021). The lectin chaperones calreticulin (CALR) and calnexin (CANX), together with their co-chaperone PDIA3, are increasingly implicated in studies of human cancers in roles that extend beyond their primary function as quality control facilitators of protein folding within the endoplasmic reticulum (ER). Led by the discovery that cell surface CALR functions as an immunogen that promotes anti-tumour immunity, studies have now expanded to include their potential uses as prognostic markers for cancers, and in regulation of oncogenic signaling that regulate such diverse processes including integrin-dependent cell adhesion and migration, proliferation, cell death and chemotherapeutic resistance (Lam and Lim, 2021). Figure 1 specifically, ELISA was adopted to determine the protein contents of HP and PDIA3 in the peripheral blood to identify the role and value of HP and PDIA3 in colorectal cancer screening, to establish statistics on relevant pathological characteristics, and to further verify the guiding value of the aforementioned two indicators (HP and PDIA3) in assessing colorectal cancer staging.

## Materials and Methods

### General Information

A selection of colorectal cancer patients, who were treated in the First Affiliated Hospital of Hebei North University from October 2020 and January 2022, were recruited in the “cancer group”. During the same period, 77 colorectal polyp patients and 36 healthy subjects were enrolled in the “polyp group” and the “group of healthy volunteers” respectively. 50 male patients and 38 female patients were included in the “cancer group”, aged from 40 to 92 and the average age being 65.8 ± 10.3; 48 male patients and 29 female patients were included in the “polyp group”, with the ages ranging from 32 to 79 and the average age being 60.5 ± 10.5; 18 men and 18 women were included in the “group of healthy volunteers”, with the ages ranging from 16 to 85 and the average age being 59.6 ± 16.2. The differences among these three groups were not statistically significant in terms of baseline data (*p* > 0.05). The Ethics Approval No. is K2022012.

### Selection Criteria

Enrollment criteria: a. The colorectal malignancy to be studied should meet the relevant diagnosis standards included in National Diagnosis and Treatment Standards for Colorectal *Cancer* (2020) (National Health Commission of the People’s Republic of China, 2020); b. The colorectal polyp to be studied should meet with the relevant diagnosis standards included in Surgery; c. The patients should not have received any anti-tumor therapy prior to enrollment.

Exclusion criteria: a. The presence of neoplastic lesions in addition to those in the colon and rectum; b. The dispensing of relevant treatment regimens; c. The loss of relevant patient information, leading to the unavailability of follow-ups to collect further prognosis and survival-rate data.

### Experimental Process

A total of 18 blood specimens (6 for each of the three groups: colorectal cancer patients, colorectal polyp patients, and healthy subjects) were selected for protein difference sequencing to explore proteins with different expressions. The collected whole blood samples were left to stand at room temperature for 2 hours, 3,000 g of which was subsequently centrifuged for 10 minutes. Following that, a volume of no less than 100 ul supernatant was taken and stored at −80°C, which was then delivered with dry ice to the Shanghai Origin-Gene Biomedical Technology Co., Ltd. for DIA quantitative proteomics testing. There are no statistical differences between “group of healthy volunteers” and “polyp group”, so we selected difference proteins from the “colorectal cancer group”and the “group of healthy volunteers”. A total of 27 differential proteins were identified; a total of 6 proteins with increased expressions and a total of 21 proteins with decreased expressions were identified in the“colorectal cancer group” and the “group of healthy volunteers”. They are Tenascin-X (TN-X), Platelet glycoprotein Ib alpha chain (GPIbA), anti-leucine-rich glioma inactivated-1 (LGI1), Melanocyte protein (PMEL), Apolipoprotein M(APOM), Haptoglobin (HP), Coagulation factor X (F10), Coagulation factor XII(F12), Apolipoprotein A-I (APOA1), Fibrinogen alpha chain (FGA), Complement C1q subcomponent subunit C(C1QC), Apolipoprotein B-100 (APOB), Immunoglobulin kappa variable 2–30(IGKV2-30), Profilin-1(PFN1), Dopamine beta-hydroxylase (DBH), Lipopolysaccharide-binding protein (LBP), Protein disulfide-isomerase A3(PDIA3), Insulin-like growth factor-binding protein complex acid labile subunit (IGFALS), Cadherin-13(CDH13), Phosphatidylinositol-glycan-specific phospholipase D (GPLD1), Prolow-density lipoprotein receptor-related protein 1 (LRP1), Apolipoprotein F (APOF), Dystroglycan (DAG1), Serum paraoxonase 3(PON3), Histone H2A type 2-C(HIST2H2AC), Protocadherin-12 (PCDH12), Protein HEG homolog 1(HEG1). We did functional enrichment about the six proteins with increased expressions on uniprot, the best choice was HP and PDIA3.

**FIGURE 2:**
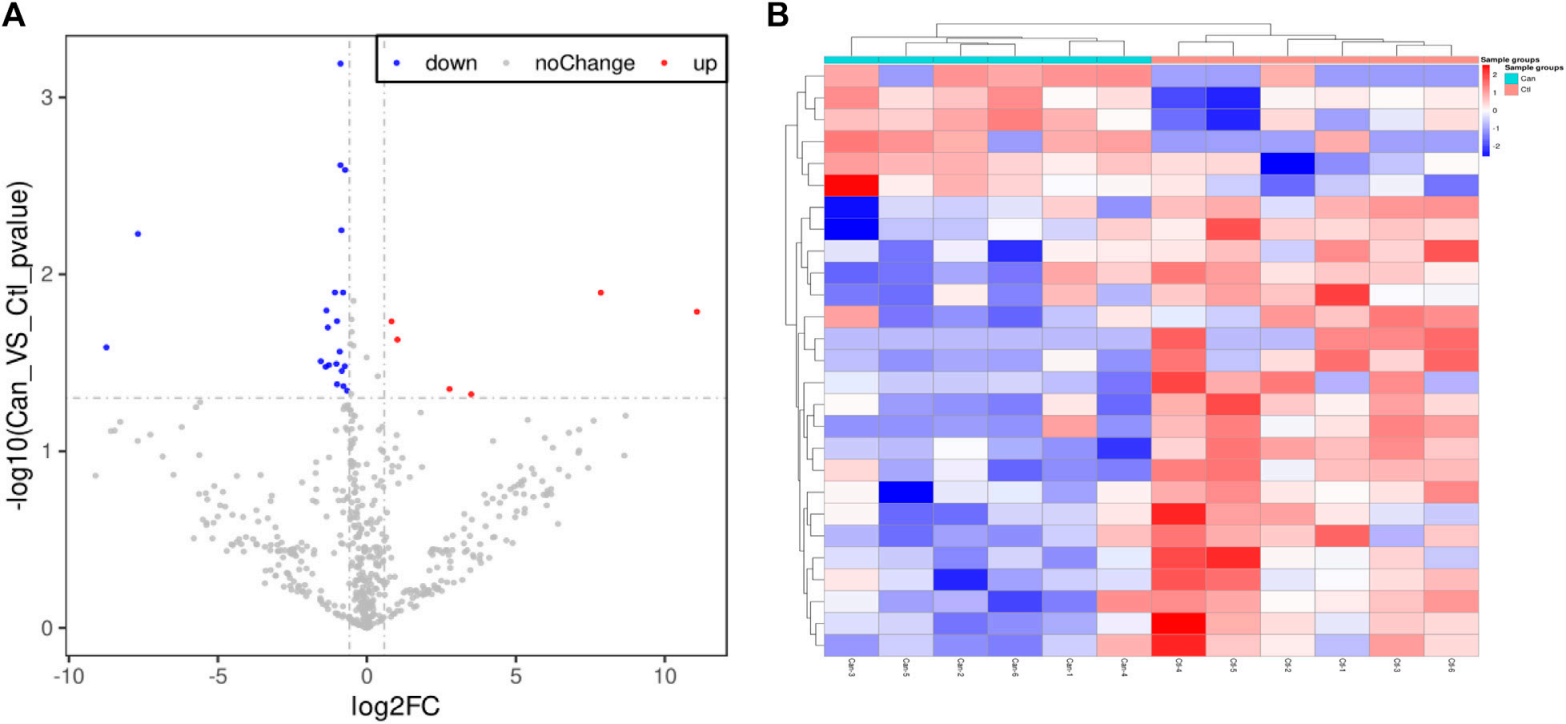
Volcano plot and Cluster heatmap. **(A)**. The volcano plot of proteome of cancer group and group of healthy volunteers, A total of 27 differential proteins were identified; a total of six proteins with increased expressions and a total of 21 proteins with decreased expressions. **(B)**. The heatmap of proteome difference with sample clustering of cancer group and group of healthy volunteers. the abscissa are the members of “cancer group” and “group of healthy volunteers”, the ordinate are the members of 27 different expressed proteins between colorectal “cancer group” and “group of healthy volunteers”.

The differences of these proteins were visualized to generate a volcano plot and a cluster heatmap (Figure 2). This study aimed to search potential biomarkers of colorectal cancer applicable to clinical use. Therefore, the focus of the study was the six proteins having increased expressions. Based on substantial literature review and analysis, HP and PDIA3 were selected from the six proteins for ELISA.

The above serum extraction method was utilized to extract an approximate amount of 0.6–0.8 ml of serum from the subsequently collected specimens. The serum was divided into three to four RNase-free tubes (each of capacity 1.5 ml) and stored in a refrigerator at −20°C. Human haptoglobin/haptoglobin ELISA kit (96T) and human PDIA3A3 ELISA kit (96T) (stored at 4°C), manufactured by Jiangsu Jingmei Biotechnology Co., Ltd., were used to test the levels of HP and PDIA3 in the serum. All procedures were in strict compliance with the instructions, with the specific steps delineated as follows: Preparation of relevant reagents prior to the experiment: dilution of standards as specified in Supplementary Tables S1,2. The specific steps are as follows: adding samples-incubation-mixing-washing-adding enzyme-incubation-washing-chromogenic reaction-stop, according to the zero setting of the blank wells, a wave of 450 nm in wavelength was adopted to measure the absorbance (OD value) of each well in the ELISA microplate. the concentration of the standard and the OD value were used to generate a quaternary linear regression equation of the standard curve; the OD value of the sample was substituted into the equation to calculate the concentration of the sample. The Figure was then multiplied by dilution multiples to yield the actual concentration of the sample. Avoid spending long time on the operation, each step is not more than 5 min.

### TNM Staging Assessment

The members of colorectal cancer patients were treated by surgery. According to pathological analysis after surgery and reference *TNM Staging for Malignant Tumors* (Rosen and Sapra, 2022). TNM staging was carried out in 88 colorectal cancer patients.

### Statistical Analysis

SPSS25.0 software was used for statistical analysis. The levels of HP and PDIA3 were represented as (‾x ± *s*); a *t*-test was used for comparison between two groups; enumeration data were expressed as percentages; a chi-square test was adopted for comparison between two groups; one-way analysis of variance was used to verify comparison among multiple groups; ROC curve was plotted to determine the area under curve (AUC). The diagnosis value of HP and PDIA3 levels to colorectal cancer patients were estimated: where AUC = 0.5–0.7, the diagnosis effect was poor; where AUC = 0.7–0.9, the effect was good; where AUC >0.9, the effect was excellent; the significance of all statistical comparisons was set to *p* < 0.05.

## Results

### Levels of HP and PDIA3

**FIGURE 3:**
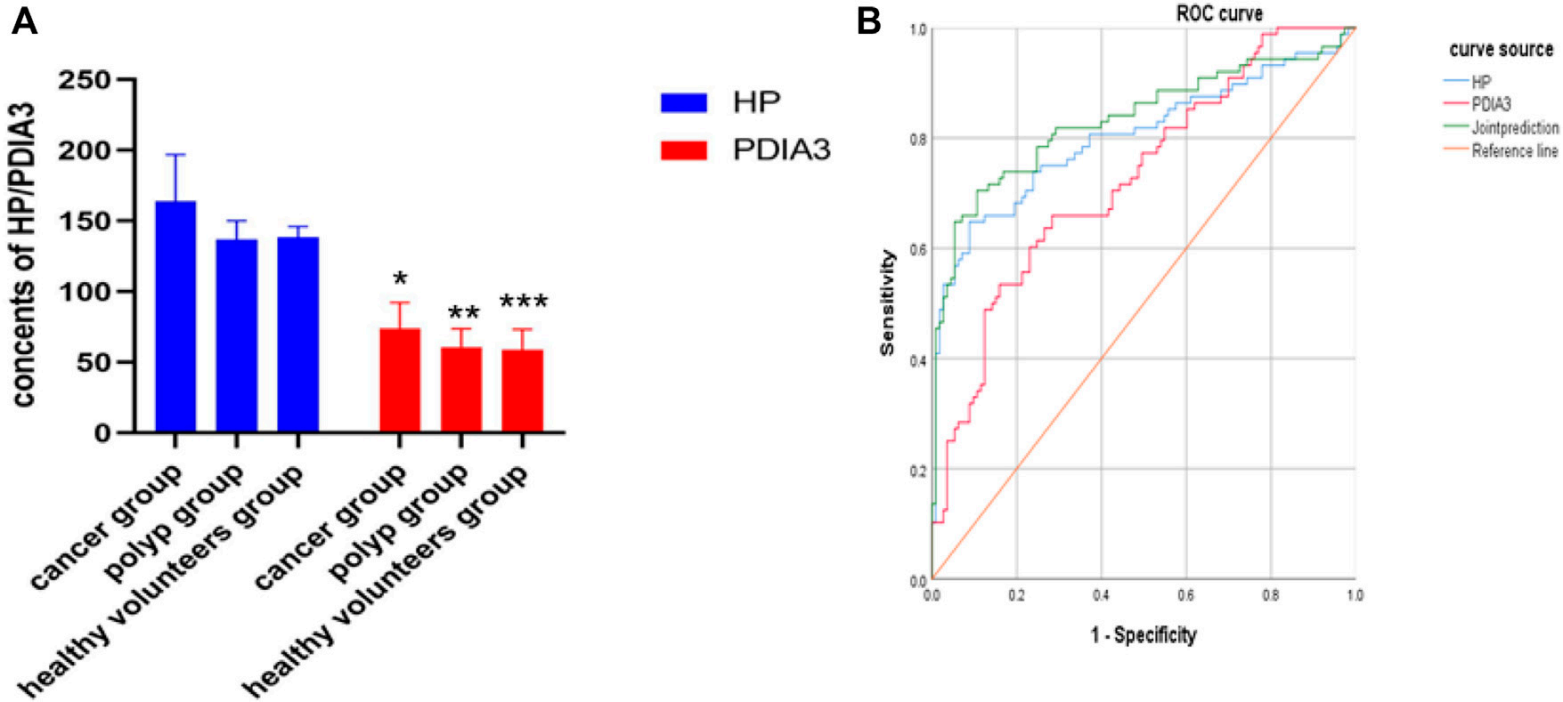
Contents of HP and PDIA3 in each group and ROC curve in which serum levels of HP and PDIA3 indicating colorectal cancer. **(A)**. In the cancer group, the average contents of HP and PDIA3 are 164 ± 33 μl/ml and 74 ± 18 ng/ml. In the polyp group, the average contents of HP and PDIA3 are 137 ± 13 μl/ml and 61 ± 13 ng/ml. In the healthy volunteers group, the average contents of HP and PDIA3 are 138 ± 8 μl/ml and 58 ± 15 ng/ml (*p* < 0.05). **(B)**. Diagnostic value of HP and PDIA3 for colorectal cancer. *vs. cancer group (*p* < 0.05), **vs. means polyp group (*p* < 0.05), *** vs. healthy volunteers group (*p* < 0.05).

In the “cancer group”, the contents of HP and PDIA3 were 164 ± 33 μl/ml and 74 ± 18 ng/ml respectively; in the “polyp group”, the above two contents were 137 ± 13 μl/ml and 61 ± 13 ng/ml respectively; in the “group of healthy volunteers”, the two contents were 138 ± 8 μl/ml and 58 ± 15 ng/ml respectively. In the “cancer group”, the average contents of HP and PDIA3 exceeded those in the “polyp group” and the “group of healthy volunteers”, showcasing statistically significant differences (*p* < 0.05); whereas the differences between the average contents in the “polyp group” and the “group of healthy volunteers” were not statistically significant (*p* > 0.05) (see Figure 3A, Supplementary Figure S1, and Table 1 for details).

**TABLE 1:** Comparison of HP and PDIA3 levels in “cancer group”, “polyp group” and “group of healthy volunteers” (*x* ± *s*). ^*^vs. cancer group vs. healthy volunteers (*p* < 0.05),^**^vs. polyp group vs. healthy volunteers (*p* > 0.05).

Group	Number of Cases	HP (μl/ml)	PDIA3 (ng/ml)
*Cancer* group	88	164 ± 33^*^	74 ± 18^*^
Polyp group	77	137 ± 13^**^	61 ± 13^**^
Healthy volunteers	36	138 ± 8	58 ± 15
*P*		0.000	0.000

### Assessing the Diagnosis Value of HP and PDIA3 Levels to Colorectal Cancer Using ROC Curve

Serum levels of HP and PDIA3 were used as test variables and colorectal cancer was adopted as state variable (1 = colorectal cancer, and 0 = colorectal polyp and group of healthy volunteers). The plotted ROC curve revealed that the cut-offs of HP and PDIA3 levels indicating colorectal cancer were 149 ug/ml and 66 ng/ml respectively (see Figure 3B and Table 2 for details).

**TABLE 2:** Value of HP and PDIA3 levels to assessment of colorectal cancer patients.

Indicator	AUC	95%CI	Standard error	*P*	Specificity	Sensitivity
HP	0.802	0.736–0.868	0.034	0.000	0.912	0.648
PDIA3	0.727	0.657–0.797	0.036	0.000	0.717	0.659
Joint prediction	0.855	0.801–0.910	0.028	0.000	0.876	0.727

### TNM Staging and Pathological Characteristics of Colon Cancer Patients

Among the 88 colorectal cancer cases, 21 were at stage I (accounting for 23.9% of the total cases), 23 at stage II (accounting for 26.1%), 30 at stage III (accounting for 34.1%), and 14 at stage IV (accounting for 15.9%). The cut-offs of HP and PDIA3 served as the baseline; high expressions were defined as above the cut-offs and low expressions were defined as below the cut-offs. The relationships between clinical characteristics and pathological characteristics and the HP and PDIA3 serum contents of colorectal cancer patients at different pathological stages were subsequently analyzed (see Table 3 for details).

**TABLE 3:** Relationships between HP and PDIA3 expressions and colorectal pathological characteristics.

Project Parameter	HP Expression		*χ* ^*2*^	*P*	PDIA3 Expression		*χ* ^*2*^	*P*
	Low	High			Low	High		
Size of tumor			2.343	0.126			3.751	0.097
<5 cm	23	31			22	32		
>5 cm	9	25			8	26		
Differentiation level			5.639	0.018			4.726	0.030
Low	2	17			2	17		
Medium, high	30	39			28	41		
Pathological staging			30.692	0.000			20.230	0.000
I + II	29	15			25	19		
III + IV	3	41			5	39		
Lymphatic metastasis			25.688	0.000			18.255	0.000
Yes	3	38			4	37		
None	29	18			26	21		

### HP and PDIA3 Serum Levels of Colorectal Cancer Patients at Different Pathological Stages

**FIGURE 4:**
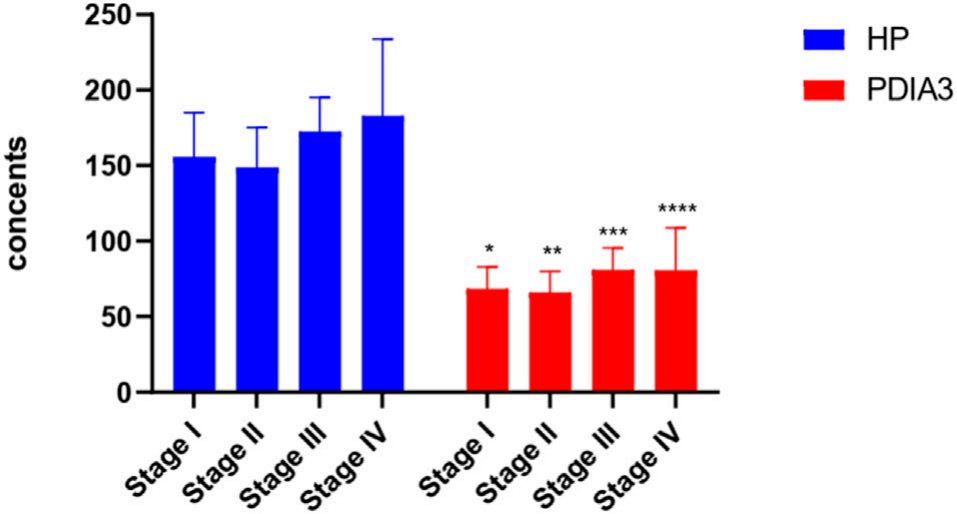
HP and PDIA3 serum contents of patients at different pathological stages. In the Stage I, the average contents of HP and PDIA3 are 156 ± 29 μl/ml and 68 ± 15 ng/ml. In the stage II, the average contents of HP and PDIA3 are 149 ± 26 μl/ml and 66 ± 14 ng/ml. In the stage III, the average contents of HP and PDIA3 are 173 ± 23 μl/ml and 81 ± 15 ng/ml. In the stage IV, the average contents of HP and PDIA3 are 183 ± 51 μl/ml and 80 ± 28 ng/ml (*p* < 0.05). *vs. Stage I (*p* < 0.05), ** vs. Stage II (*p* < 0.05), ***vs. Stage III (*p* < 0.05), ****vs. Stage IV (*p* < 0.05).

HP and PDIA3 levels of stage-I patients were 156 ± 29 μl/ml and 68 ± 15 ng/ml respectively; those of stage-II patients were 149 ± 26 μl/ml and 66 ± 14 ng/ml respectively; those of stage-III patients were 173 ± 23 μl/ml and 81 ± 15 ng/ml respectively; those of stage-IV patients were 183 ± 51 μl/ml and 80 ± 28 ng/ml respectively. The above figures account for the differences in the expression levels of HP and PDIA3 of patients at different stages (*p* < 0.05, Figure 4, Supplementary Figure S2, and Table 4).

**TABLE 4:** Comparison of HP and PDIA3 levels in patients with different TNM stages of colorectal cancer (*x* ± *s*). ^*^vs. Stage I (*p* > 0.05),^**^vs. Stage III (*p* > 0.05).

TNM Staging	Number of Cases	HP (μl/ml)	PDIA3 (ng/ml)
Stage I	21	156 ± 29	68 ± 15
Stage II	23	149 ± 26^*^	66 ± 14^*^
Stage III	30	173 ± 23	81 ± 15
Stage IV	14	183 ± 51^**^	80 ± 28^**^
*P*		0.004	0.005

### Value of CEA and CA199 Levels to Assessment of Colorectal Cancer Patients

**FIGURE 5:**
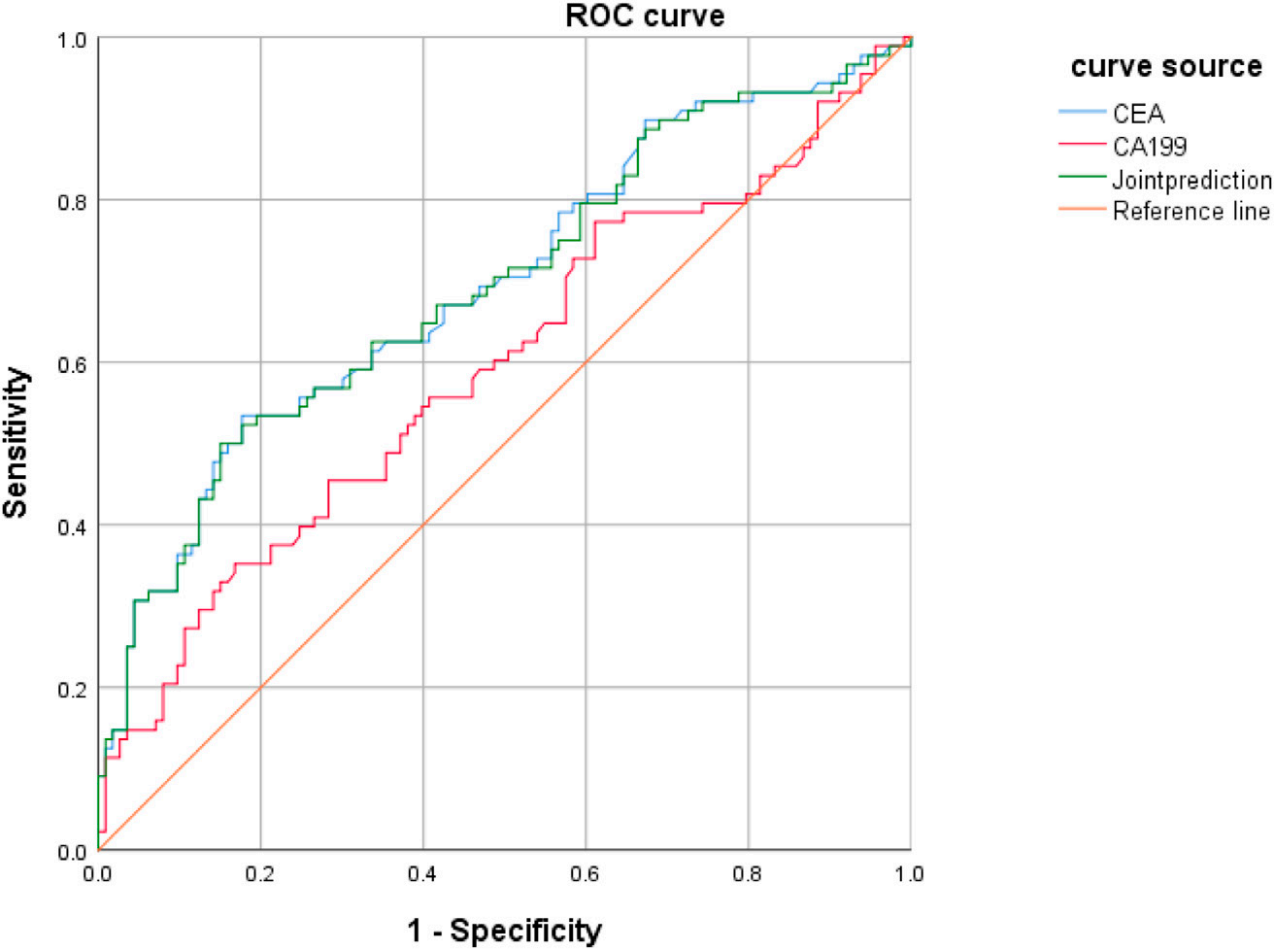
ROC curve in which CEA and CA199 levels indicating colorectal cancer.

CEA and CA199 levels were used as test variables and colorectal cancer was adopted as state variable (1 = colorectal cancer, and 0 = colorectal polyp and group of healthy volunteers) to plot an ROC curve. The diagnosis accuracy of colorectal cancer through CEA was 69.9%, that through CA199 was 61.4%, and that through joint testing was 71% (Figure 5 and Table 5).

**TABLE 5:** Value of CEA and CA199 levels to assessment of colorectal cancer patients.

Indicator	AUC	95%CI	Standard error	*P*	Specificity	Sensitivity
CEA	0.699	0.625–0.773	0.38	0.001	0.823	0.534
CA199	0.614	0.535–0.693	0.40	0.006	0.832	0.375
Joint test	0.710	0.638–0.78	0.37	0.001	0.85	0.523

### Value of HP and PDIA Levels to the Assessment of Colorectal Cancer Stages

**FIGURE 6:**
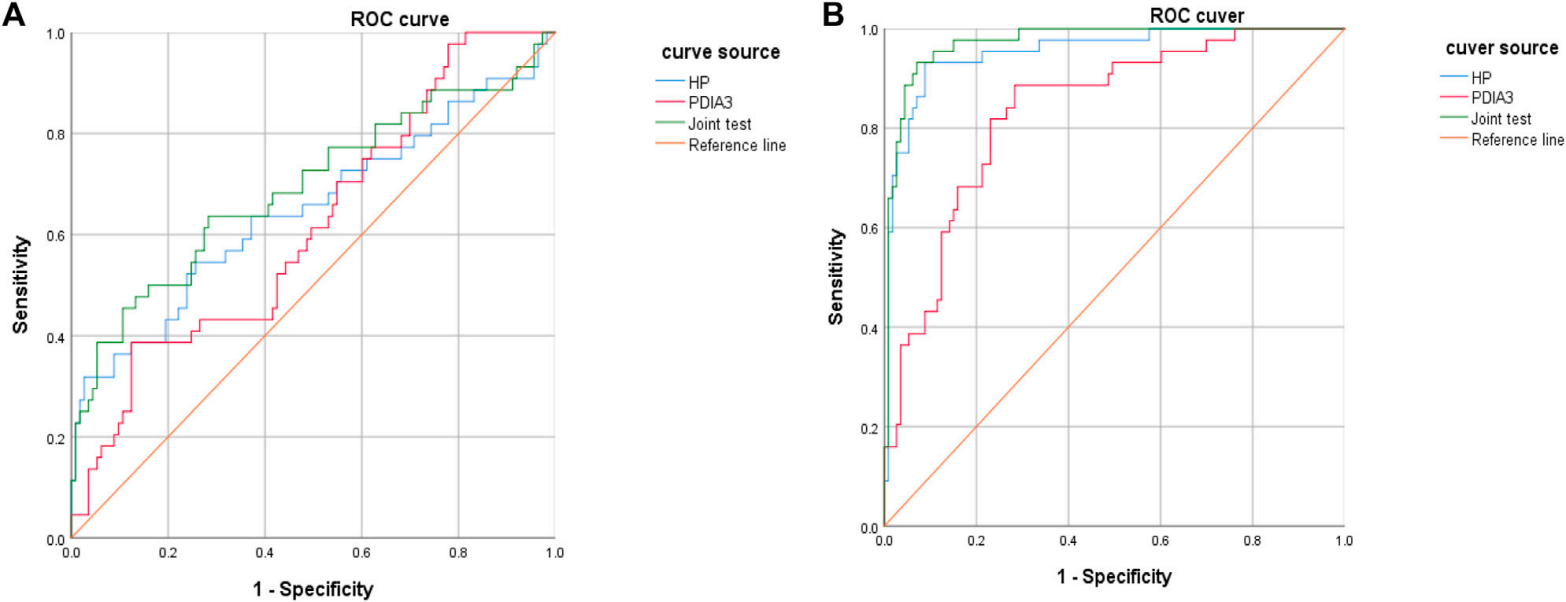
Diagnosis value of HP and PDIA3 to early colorectal cancer and progressive stage. **(A)**. Low diagnosis accuracy of early colorectal cancer. **(B)**. Great assessment value to pathological staging of colorectal cancer.

HP and PDIA3 levels were used as test variables and colorectal cancer was adopted as the state variable (1 = colorectal cancer (including early stage and progressive stage), and 0 = colorectal polyp and group of healthy volunteers) to plot an ROC curve. The diagnosis accuracy of early and progressive colorectal cancers through HP were 65 and 95.4% respectively, the accuracy through PDIA3 were 61.8 and 83.6% respectively, and that through joint testing were 69.3 and 97.3% respectively (see Figure 6A, Figure 6B, Table 6, and Table 7).

**TABLE 6:** Value of HP and PDIA3 levels to assessment of patients with early colorectal cancer.

Indicator	AUC	95%CI	Standard error	*P*	Specificity	Sensitivity
HP	0.650	0.544–0.756	0.054	0.004	0.973	0.318
PDIA3	0.618	0.521–0.714	0.049	0.022	0.876	0.386
Joint test	0.693	0.591–0.794	0.052	0.000	0.717	0.636

**TABLE 7:** Value of HP and PDIA3 levels to assessment of colorectal cancer patients at progressive stage.

Indicator	AUC	95%CI	Standard error	*P*	Specificity	Sensitivity
	0.954	0.919–0.990	0.018	0.000	0.912	0.932
PDIA3	0.836	0.768–0.903	0.034	0.000	0.717	0.886
Joint test	0.973	0.951–0.995	0.011	0.000	0.929	0.932

## Discussion

Colorectal cancer (CRC) alone accounts for ∼10% of total new global cases and poses an over 4% lifetime risk of developing cancer (Rada et al., 2021; Saeed et al., 2021; Si et al., 2021; Weng and Goel, 2022). Presently, the common approaches for colorectal cancer screening mainly consist of invasive examinations (sigmoidoscopy, colonoscopy, barium enema, etc.), non-invasive examinations (stool examination, etc.), and radio-assay (such as fecal occult blood test, fecal immunochemical test (FIT), multi-target FIT DNA test, and tumor marker assay) (Issa and Noureddine, 2017).

Currently, fecal occult blood testing is the most extensively used screening method for colorectal cancer. However, due to its low sensitivity and specificity (specifically, its lack of specificity to the hemoglobin in the feces and its susceptibility to drugs and food containing ferrous iron) this test method is considered obsolete and has been replaced by fecal immunochemical test (FIT). Nevertheless, the presence of hysteresis arising from immune hook effect and antigen excess associated with IFT is likely to entail false negatives during clinical use. Multi-target FIT DNA testing (FIT-DNA) has good sensitivity and specificity to colorectal cancer. However, it is difficult to include in Chinese clinical practice due to the high cost screening and the low acceptability to the general population and colonoscopy remains the gold diagnosis standard for colorectal cancer. Previous research suggests that the early diagnosis and excision of colorectal lesions through colonoscopy can reduce the incidence of colorectal cancer. However, this investigation requires bowel cleaning thereby entailing some invasion and risks. Accordingly, the population compliance is low which substantially limits the diagnosis rate. Research reveals that the participation rates of colonoscopy and fecal immunochemical testing are 42.5 and 94.0% respectively (Chen et al., 2020). Given the current situation of the aforementioned colorectal malignancy screening methods, it is imperative to establish screening methods for colorectal cancer with high sensitivity, strong specificity, and good compliance.

Through quantitative proteomics testing and screening, two types of proteins were selected for research, namely HP and PDIA3. HP is an acid α2 glycoprotein present in the serum and body fluids of mammalian species including humans. Gene expressions of HP were successively discovered in the liver, heart, spleen, and other organs. Despite the presence of three HP genotypes: HP1-1, HP2-1, and HP2-2, all of which are associated with susceptible infectious diseases, atherosclerosis, autoimmune disorders, and vascular occlusive diseases (Goldenstein et al., 2012; Andersen et al., 2017; Simon et al., 2020). A study on HP informatics (Naryzhny and Legina, 2021) has revealed that HP is a stable hydrophilic protein, composed of 406 amino acids, with a long half-life of about 30 h. HP has two peptide chains, namely α and β, which are connected by a disulfide bond. Fucosylation, characterized by abnormal β peptide chain, is closely associated with cancer. Being an important oligosaccharide modification mode, fucosylation mainly occurs in tumor and inflammatory responses and exists in various biological processes such as origin, differentiation, and growth of cells, as well as formation and metastasis of infected tumors (Jeong et al., 2020). Previous studies demonstrate that HP expressions are higher in the serum of patients with liver cancer, lung cancer, ovarian cancer, or other cancers than in the serum of healthy subjects. Furthermore, HP expressions combined with other serum markers, would be of reference value to the early diagnosis of relevant cancers and of some guiding significance in terms of treatment and prognosis (Naryzhny et al., 2021).

PDIA3 is a member of the protein disulfide isomerase (PDI) family that is coded by genes. It serves both as an enzyme, and a chaperone. As a multifunctional protein, PDIA3 exists extensively in endoplasmic reticulums of cells. It is known that functional and viral infections (Schelhaas et al., 2007) as well as cell proliferation are connected to apoptosis (Panaretakis et al., 2008). PDIA3 exists widely in various human tissues and its expressions vary. Moreover, the expression dysregulation of PDIA3 is associated with relevant diseases. Therefore, an increasing number of studies use PDIA3 as a biomarker to assess the diagnosis and prognosis of some diseases. The expression dysregulation of PDIA3 has been assessed in several gastrointestinal cancers, and PDIA3 over-expressions in gastric carcinoma and colon cancer have been proven (Ren et al., 2006; Yang et al., 2018; Shimoda et al., 2019). Furthermore, PDIA3 can alter the chemotherapy resistance and the radiotherapy sensitivity of gastrointestinal cancers by inhibiting or activating other proteins (Choe et al., 2015). Moreover, PDIA3 features the ability to promote the proliferation of cancer cells and playing a part in cancer progression. Even though relevant mechanisms of action are still under investigation, PHIA3, as a novel anti-cancer marker, has promising application prospects.

The results of this study are as follows. Serum levels of HP and PDIA3 were significantly higher in the “cancer group” than in the “polyp group” and the “group of healthy volunteers.” However, there were no significant differences between the “polyp group” and the “group of healthy volunteers”. There were also significant differences in HP and PDIA3 levels between colorectal cancer patients at an early stage and those at the progressive stage, indicating that HP and PDIA3 expressions were high in malignant tumors and would continuously increase alongside disease progression. It was found through the ROC curve that the accuracy of individual or joint testing of HP and PDIA3 markedly exceeded that of traditional CEA and CA199. Further stratification analysis revealed that HP and PDIA3 contents were of great assessment value to pathological staging of colorectal cancer, particularly to the progressive stage. The diagnosis accuracy of early colorectal cancer by joint testing was 69.3% and the diagnosis accuracy of progressive colorectal cancer through this test reached a peak of 97.3%. Early lesions differ slightly from normal tissues and may account for the lower diagnosis accuracy of early colorectal cancer.

The experiment also has deficiencies such as the relatively small sample volume, the absence of comparison regarding the serum contents of these two proteins of patients before and after surgery, the absence of survival follow-ups leading to inability to probe deep the effect of the two protein contents on prognosis, and failure to further explore the mechanisms of action of HP and PDIA3. As follow-through, cooperation featuring multiple centers and large samples could be implemented to deliver authentic and accurate conclusions.

## Data Availability

The original contributions presented in the study are included in the article/Supplementary Material, further inquiries can be directed to the corresponding authors.

## Abbreviations

HP, Haptoglobin; PDIA3, Protein disulfide-isomerase A3; CRC, Colorectal cancer; ELISA, Enzyme linked immunosorbent assay; ROC curve, Receiver operating characteristic curve; AUC, Area under the cure; CEA, Carcinoembryonic antigen; CA199, Carbohydrate Atigen 19–9; FIT, fecal immunochemical test.
