# Supplementary figures and images for: Clinical Significance of Serum Haptoglobin and Protein Disulfide-Isomerase A3 in the Screening, Diagnosis, and Staging of Colorectal Cancer
Source: Front Pharmacol. 2022 Jul 4;13:935500. doi: 10.3389/fphar.2022.935500 (PMC9290321; doi:10.3389/fphar.2022.935500)

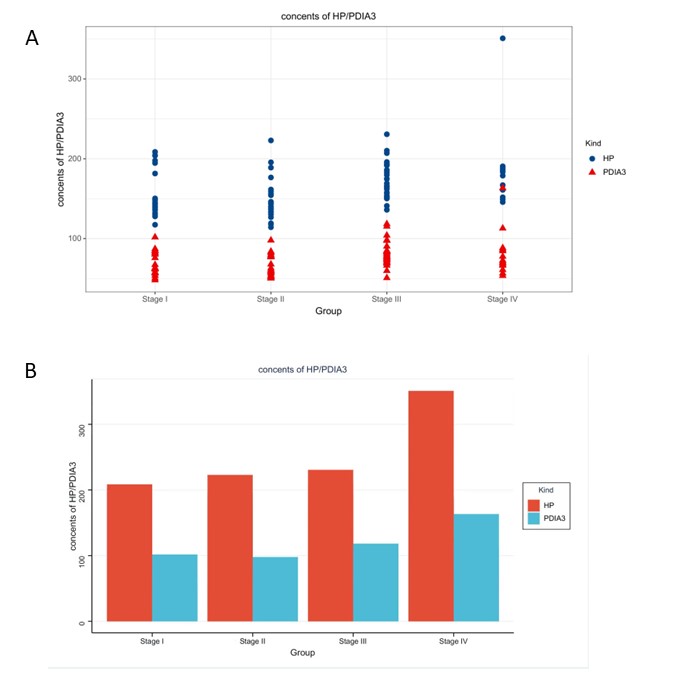

Supplement: Supplementary file 1 [file Image2.jpg]

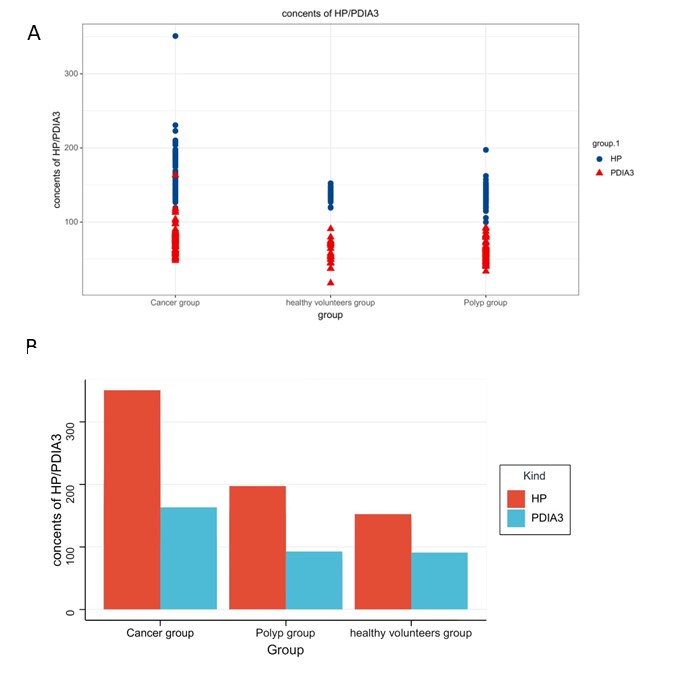

Supplement: Supplementary file 4 [file Image1.jpg]
