# Supplementary material for: Clinical Significance of Serum Haptoglobin and Protein Disulfide-Isomerase A3 in the Screening, Diagnosis, and Staging of Colorectal Cancer
Source: Front Pharmacol. 2022 Jul 4;13:935500. doi: 10.3389/fphar.2022.935500 (PMC9290321; doi:10.3389/fphar.2022.935500)
Supplement: Supplementary file 3 [file Table2.docx]

**Suppl Table 2. Dilution method for HP (dilution with five different standard).**

| 240 μl/ml | No. 5 standard | Adding 150 ul of the original standard into 150ul of the diluted standard |
| --- | --- | --- |
| 120 μl/ml | No. 4 standard | Adding 150 ul of the original standard into 150ul of the diluted standard |
| 60 μl/ml | No. 3 standard | Adding 150 ul of the original standard into 150ul of the diluted standard |
| 30 μl/ml | No. 2 standard | Adding 150 ul of the original standard into 150ul of the diluted standard |
| 15 μl/ml | No. 1 standard | Adding 150 ul of the original standard into 150ul of the diluted standard |
